# A Non-Enzymatic and Label-Free Fluorescence Bioassay for Ultrasensitive Detection of PSA

**DOI:** 10.3390/molecules24050831

**Published:** 2019-02-26

**Authors:** Yujie Sun, Chenyun Wang, Hong Zhang, Yulin Zhang, Guojun Zhang

**Affiliations:** 1 School of Laboratory Medicine, Hubei University of Chinese Medicine, 1 Huangjia Lake West Road, Wuhan 430065, China; fredsunsun@hotmail.com (Y.S.); 1731208003@stmail.hbtcm.edu.cn (C.W.); 2 Teaching and Research Office of Forensic Medicine, Hubei University of Chinese Medicine, 1 Huangjia Lake West Road, Wuhan 430065, China; redguo@hotmail.com

**Keywords:** prostate specific antigen, hybridization chain reaction, label-free, bioassay

## Abstract

The early diagnosis of prostate cancer is very vital for the improvement of patient survival chances. The content of prostate specific antigen (PSA) in serum is closely related to the status of the prostate cancer. We report a fluorescence bioassay, capable of detecting PSA in a non-enzymatic and label-free manner. PSA gives rise to the structural change of a hairpin, consequently triggering the hybridization chain reaction and forming a long-nicked double-helix, which is not adsorbed by graphene oxide. GelRed, as the signal indicator, then binds with dsDNA molecule, thereby producing the fluorescence. The established bioassay has the merits of simple operation, favorable cost-to-benefit ratios, good stability, and specificity. Moreover, the detection limit of this assay is as low as 10 pg/mL, and the linearity range is wide—from 100 pg/mL to 200 ng/mL. At the same time, this bioassay can realize the detection of PSA in biological samples (human serum, saliva, and urine). Therefore, the bioassay provides a potential means for the early diagnosis of prostate cancer.

## 1. Introduction

Prostate cancer (PCa) has become one of the most common tumors among men, especially in elderly males [1,2,3]. Early diagnosis plays a very crucial role in improving survival chances of PCa patients [2]. The traditional clinical diagnostic techniques, such as transrectal ultrasonography, digital rectal examination (DRE), computed tomography scanning, and magnetic resonance imaging, are usually complicated, time-consuming, and need to be performed by skilled professionals. Moreover, most of these techniques cannot realize a diagnosis of PCa cases in their initial stages [4,5]. There is an increasing demand for cost-effective, simple, reliable, and rapid methods for the early diagnosis of PCa. Prostate specific antigen (PSA) is a 33–34 kDa glycoprotein, secreted mainly by the prostate gland, and is the most effective serum marker for diagnosing PCa [6]. Intensive studies about the detection of PSA content for early diagnosis of PCa have become the current mainstream research direction.

Aptamers are synthetic oligonucleotides (DNA or RNA) which are selected, in vitro, according to their ability to bind to targets (including proteins, small molecules, and cells) [7,8,9]. Aptamers have multiple advantages over antibodies, such as simple synthesis, convenient modification, good stability, and low cost [10,11]. As a result, aptamers are increasingly being utilized as recognition elements in the bioassay platforms, including colorimetric, electrochemical, field effect transistor, Raman spectroscopy, and fluorescent [11,12,13,14]. Aptamer-based fluorescence methods, for their simple operation, fast response, and low cost, have gained particular attention for the detection of disease-related biomarkers [15,16]. However, most of the aptameric assays not utilizing signal amplification strategies cannot meet the requirements for early diagnosis of tumor patients. To solve this problem, various aptamer-based signal amplification strategies have been employed, including nicking endonuclease, DNA rolling circle amplification (RCA), enzyme-mediated DNA chain elongation, and so on [17]. Although these methods have made significant improvements to the sensitivity of fluoroimmunoassays, they all require the assistance of protein enzymes. However, enzyme activities are always environment-dependent. In other words, the enzyme activities are varying if the surroundings undergo even minor changes [16]. There is, thus, an adverse effect on the reproducibility of the established methods.

A hybridization chain reaction (HCR) is a triggered self-assembly process, powered by the free energy of base pair formation and leading to the polymerization of oligonucleotides into a long-nicked dsDNA molecule. As an enzyme-free signal amplification strategy, HCR possesses many advantages, such as mild condition requirements, strong environmental tolerance, and good reproducibility [18,19]. Among these HCR-based fluorescence methods, fluorescence-labeled hairpin probes have usually been used as the signal indicators [20]. However, fluorescence-labeled probes also suffer from problems, including high cost, low yield, and a complex purification process [21,22].

GelRed is an intercalating nucleic acid stain, usually used in molecular biology for gel electrophoresis [23,24]. Its own fluorescence can be ignored, whereas it has a strong fluorescence intensity when bound with nucleic acid. Moreover, it is less toxic and more sensitive, compared with other DNA-intercalating reagents [24]. However, GelRed has a lack of selectivity toward ssDNA and dsDNA.

Graphene oxide (GO), a single-atom thick, two-dimensional carbon nanomaterial with extraordinary electronic, mechanical, and optical properties, as well as good water-solubility, has been widely applied in biological and biomedical areas [6,25,26]. GO has received more attention as a material in fluorescence methods due to its important characteristics, such as being a highly efficient fluorescence quencher and having high affinity to ssDNA but weak affinity to dsDNA. The combination of GO with HCR strategies used for the detection of disease-related biomarkers has also been reported [27,28]. However, the methods utilized the labeled fluorescent probe.

Inspired by these general studies, we present an enzyme-free (as well as label-free) fluorescence assay for the detection of PSA, by combination of GO with HCR and GelRed. H1 was composed of the PSA aptamer sequence, as well as the HCR triggering sequence. In the presence of PSA, the hairpin structure of H1 opens up and the initiation sequence is exposed to H2 and open up the hairpin structure of H2, then triggers the HCR reaction. The GelRed binds with the HCR product (long-nicked dsDNA molecules), irradiating a strong fluorescence. Even though GO is added, the change in fluorescence intensity is very small, due to the weak affinity between GO and the HCR product [29]. However, HCR cannot be triggered in the absence of PSA. In such a case, H1, H2, and H3 are absorbed by GO and the fluorescence signal is very weak. The research results indicate that the established assay can realize a highly sensitive and specific detection of PSA. The established platform will offer a valuable detection tool for the early diagnosis of PCa.

## 2. Results and Discussion

### 2.1. Principle and Feasibility of the Assay

**Figure 1:**
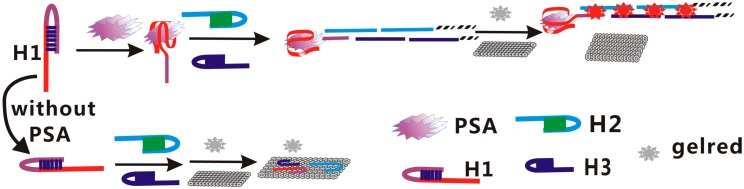
Schematic illustration of the fluoroimmunoassay detection of prostate specific antigen (PSA) based on the combination of target-triggered hybridization chain reaction, GelRed and graphene oxide (GO).

The principle diagram is shown in Figure 1. This assay contains 3 ssDNA: H1, H2, and H3. As shown in Figure 1, the H1 sequence was composed of two parts: The red part was the PSA sequence, and the other part (lilac) contained a trigger sequence of HCR reaction which is complementary with part of H2 on the 5′ terminal, and 9 successive adenine bases to reduce any possible steric hindrance effects. In the absence of PSA, these 3 ssDNAs can coexist in solution, in the form of hairpins. When GelRed was added, a strong fluorescence signal emerged; but the fluorescence signal became very weak after adding GO. This is ascribed to the fact that the 3 ssDNAs were adsorbed on the GO surface through their sticky ends and loops. In the presence of PSA, the PSA can bind with the aptamer sequences of H1 to form a PSA–aptamer complex. Hence, the trigger sequence was exposed and hybridized with the sticky end of H2, resulting in an opening up of the hairpin of H2. The formed exposed sequence of H2 hybridized with the sticky end of H3, leading to an opening up of the hairpin of H3. The hybridization processes are repeated alternatively and a long-nicked dsDNA molecule forms. When GelRed and GO are added, a strong fluorescence signal appears, because the long-nicked dsDNA molecule has very weak affinity with GO. In such a way, the detection of PSA can be realized.

**Figure 2:**
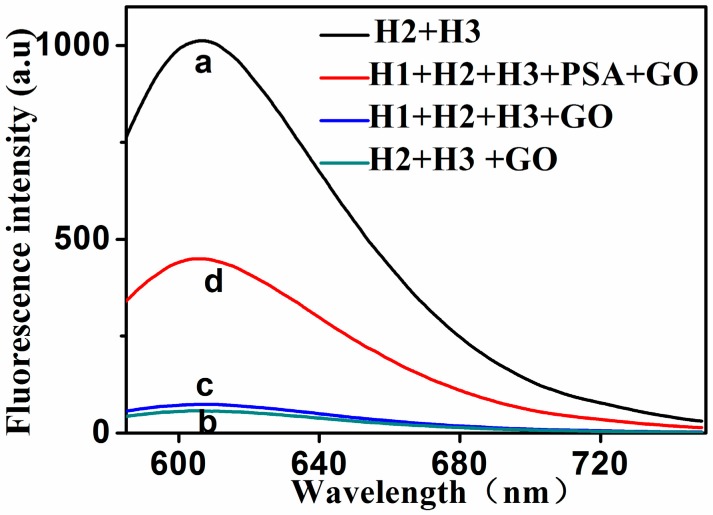
The typical fluorescent emission spectra of different mixtures solution. The mixture solutions, for curves a to d, are: (**a**) H2 + H3; (**b**) H2 + H3 + GO; (**c**) H1 + H2 + H3 + GO; and (**d**) H1 + H2 + H3 + PSA + GO. The concentrations of H1, H2, H3, GO and target (PSA) were 20 nM, 30 nM, 30 nM, 15 μg·mL^−1^ and 5 ng·mL^−1^, respectively.

In order to verify the feasibility of the proposed sensing system for PSA assay, the fluorescence signals of PSA measurements in the presence of PSA, as well as a series of control experiments, are depicted in Figure 2. GelRed was added in all measurements. The mixture solution of H2 and H3 had a relatively strong fluorescence signal (Figure 2a). This is because the GelRed can chelate with H2 and H3 to form a strong fluorescent signal. When the GO was added to the mixture solution of H2 and H3, the fluorescence intensity became very weak (Figure 2b). This is because H2 and H3 were both absorbed onto the surface of GO. The fluorescence signal was still very weak, even if the H1 was added in the presence of GO (Figure 2c). This indicates that H1, H2, and H3 could co-exist in solution in the form of hairpins, and that they were all absorbed onto the surface of GO. However, when 5 ng/mL of PSA was added into the mixture solution of H1, H2, and H3, the fluorescence intensity was significantly enhanced, even in the presence of GO (Figure 2d), indicating that PSA specifically bound to H1, triggering the HCR reaction. The fluorescence intensity was 449 with 5 ng/mL PSA, and the fluorescence intensity was 70 without PSA. The fluorescent emission spectra of the solution of (H1 + GO) and (H1 + PSA + GO) are given in the Supporting Material (Figure S3). These results confirm the feasibility of the proposed fluorescence bioassay for PSA detection.

### 2.2. Optimization of Reaction Conditions

**Figure 3:**
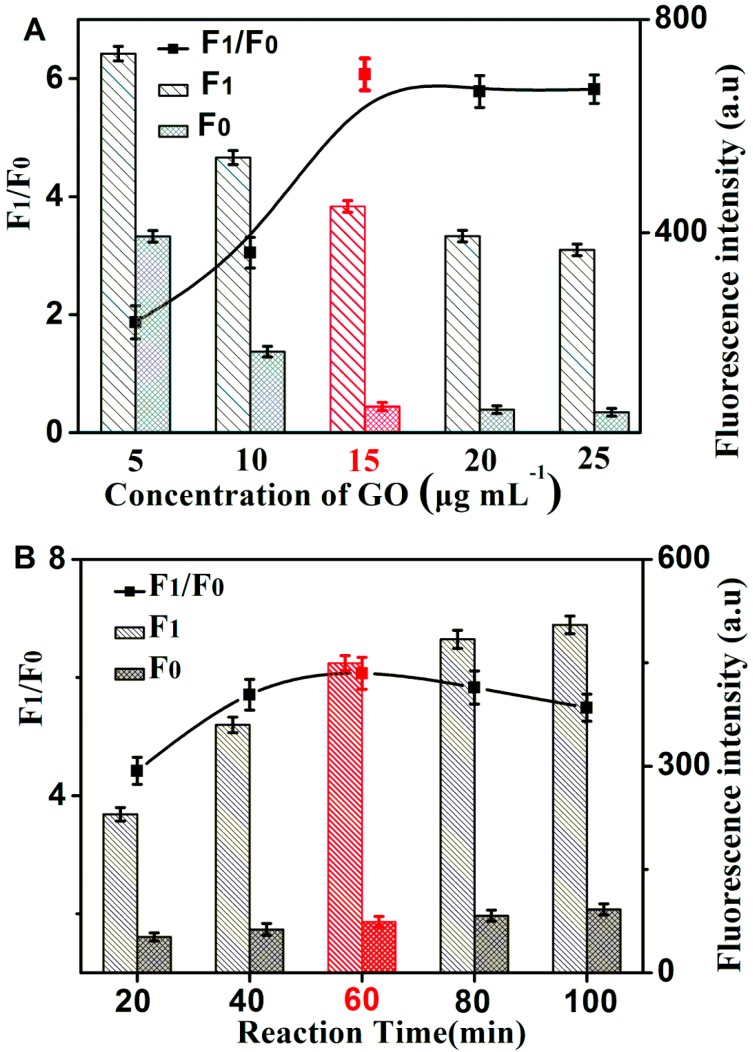
(**A**) The fluorescence intensity of this assay under different GO concentrations. (**B**) The effect of HCR time on the fluorescence responses. F_1_ with 5 ng·mL^−1^ of PSA; both H2 and H3 were 30 nM.

The fluorescence signal in the presence of H1 H2, H3, and GelRed can be effectively quenched by GO, which is vital in the design of the HCR/GO/GelRed assay. The relationship between the fluorescence change and the concentration of GO is shown in Figure 3A. The value of F_1_ and F_0_ obviously decreased with an increase in the concentration of GO. The value of F_1_/F_0_ was at maximum value when 15 μg/mL GO was added. This value was, therefore, selected to be the optimal GO concentration. The reaction time of HCR also had an important effect on fluorescence intensity. The values of F_1_ and F_0_ increased with longer reaction times (as shown in Figure 3B). The maximum F_1_/F_0_ value was obtained when reaction time was 60 min. So, 60 min was selected to be the optimal HCR reaction time for the rest of the experiments. F_1_ and F_0_ is the fluorescence values at 606 nm in the presence and absence of PSA, respectively.

Besides the aforementioned conditions, pH, HCR reaction temperature, and the concentrations of H1, H2, and H3 may also affect the sensitivity. The corresponding experimental results of pH, HCR reaction temperature, and the concentrations of H1, H2, and H3 are shown in the Supplementary Materials (Figures S1 and S2). The optimal pH value was selected as 7.4. The HCR reaction temperature was chosen to be 37 °C. The optimal concentration of 30 nM was selected for H2 and H3, and 20 nM was confirmed as the optimal reaction concentration for H1.

### 2.3. Sensitivity and Specificity for PSA Detection

**Figure 4:**
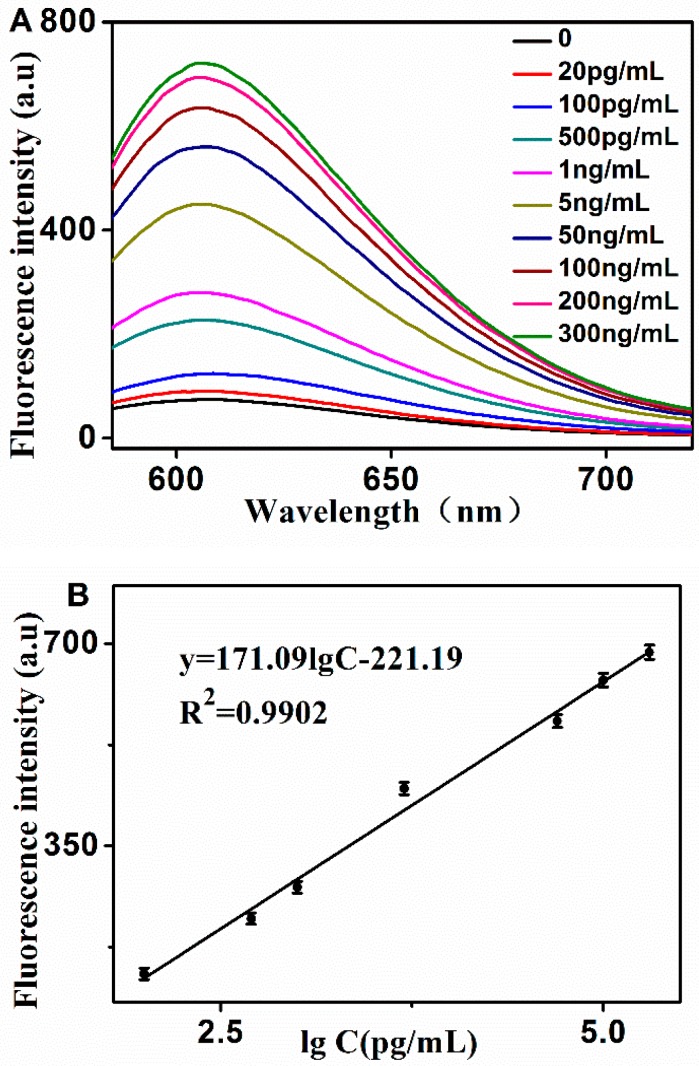
(**A**) Fluorescence emission spectra in the presence of PSA with different concentrations. From bottom to top: 0 to 300 ng·mL^−1^, respectively. (**B**) The relationship curve of the fluorescence response to PSA concentration. The experimental conditions: H1, 20 nM; H2, 30 nM; H3, 30 nM; GO, 15 μg·mL^−1^.

Under the optimal experimental conditions, the fluorescence emission spectra of the assay at various PSA concentrations were investigated. As shown in Figure 4A, the fluorescence signal increased dynamically with the increase of PSA concentration. Figure 4B depicts the relationship between the fluorescence intensity and the PSA concentration. As shown in the inset of Figure 4B, the value of F_1_ was proportional to the logarithm of the PSA concentration, ranging from 0.1–200 ng/mL. The linear regression equation was F_1_ = 171.09 × C − 221.19 with a correlation coefficient of 0.9902, and the detection limit was determined as 3 times the signal-to- noise ratio, and was calculated to be 10 pg/mL (C is the concentration of PSA). Comparing with other (previously reported) methods for the detection of PSA, the low detection limit of this assay was comparable to or even lower (as shown in Table 1). The established method utilized the aptamer to recognize the PSA; this is a more cost-effective comparison, utilizing the antibody as the recognized element. HCR is an enzyme-free signal amplification strategy, and so this strategy has a strong environmental tolerance. However, the reaction time needs 1 h, which is longer than the method shown in Table 1. The high sensitivity of the established method may be ascribed to the following factors: (1) HCR is a highly efficient signal amplification method, in which a target molecular can trigger the formation of multiple signal responses; (2) GO has a high affinity toward ssDNA and weakly affinity towards the long-nicked dsDNA molecule, resulting in a high signal-to-noise ratio; and (3) GelRed, when bound with nucleic acid, can induce a strong fluorescence signal, also producing a high signal-to-noise ratio.

**Figure 5:**
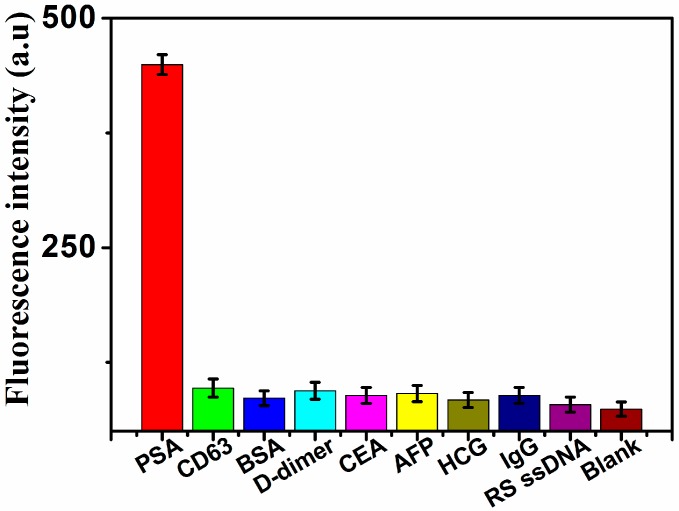
Fluorescence response of the assay in the presence of PSA (5 ng·mL^−1^), CD63 (50 ng·mL^−1^), BSA (50 ng·mL^−1^), D-dimer (50 ng·mL^−1^), CEA (5 ng·mL^−1^), AFP (50 ng·mL^−1^), HCG (50 ng·mL^−1^), IgG (50 ng·mL^−1^), RS ssDNA (5 nmol·mL^−1^), and blank control, respectively.

In addition, the specificity of the assay was also investigated by adding seven different relevant control proteins, CD63, BSA (Bovine Serum Albumin), Carcinoembryonic antigen (CEA), alpha feto-protein (AFP), human chorionic gonadotropin (HCG), immunoglobulins G (IgG) and D-dimer as well as random sequence ssDNA (RS ssDNA). As shown in Figure 5, a negligible change in the fluorescence signal was exhibited in the presence of CD63, BSA, CEA, AFP, HCG, D-dimer and RS ssDNA, compared with the blank control. However, a significant increase of fluorescence intensity was obtained in the presence of PSA, indicating that this method exhibits high specificity for PSA detection.

### 2.4. Determination of PSA in Real Samples

**Figure 6:**
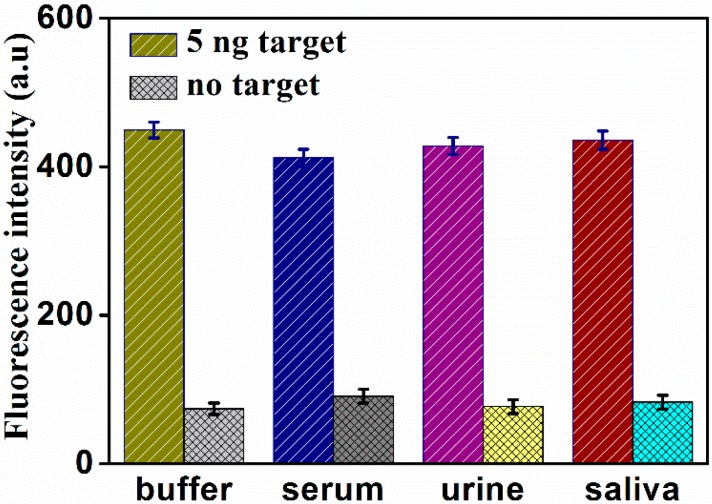
Fluorescence response of the assay for detection of PSA in buffer and various blank biological samples (human serum, urine, and saliva).

In order to evaluate the performance of this bioassay for the detection of PSA in practical samples, two different concentrations of PSA were added into blank biological samples, including human serum, urine, and saliva. All spiked samples were diluted to 10%. As shown in Figure 6, a distinct enhancement of the fluorescence intensity was observed in various biological samples in the presence of 5 ng/mL PSA, compared with blank biological samples. Meanwhile, the difference of the signal between the buffer and diluted human fluid was within 20%. The results of 0.5 ng/mL PSA are shown in Figure S4. These results indicate that the proposed assay was feasible for analyzing PSA in biological samples.

## 3. Experimental Section

### 3.1. Reagents and Materials

The GelRed (1 × 10,000) was purchased from the Solarbio Science & Technology Co., Ltd. (Beijing, China, www.solarbio.com). The H1 (5′-AGC TTT AAT TAA CAT GTC CGAC AAA AAA AAA TTA ATTA AAGCT CGC CAT CAA ATA GC-3′), H2 (5′-AGC TTT AAT TAA CAT GTC CGA CTA ATT AAA GCT AGT CGA TGC TA-3′), H3 (5′-GTC GGA CAT GTT AAT TAA AGC TTA GCA TCG ACT AGC TTT AAT TA-3′), and random sequence ssDNA (RS ssDNA) (5′-TCATGACTTATCACAGTAGTAA-3′) were synthesized and purified using HPLC by the Sangon Biotechnology Co. Ltd. (Shanghai, China, www.sangon.com). The PSA, BSA, CD63 Carcinoembryonic antigen (CEA), alpha feto-protein (AFP), human chorionic gonadotropin (HCG), immunoglobulins G (IgG), and D-dimer were purchased from Cusabio Biotech Co. Ltd. (Wuhan, China, www.cusabio.cn). GO was synthesized, according to the previous report [35,36]. The other reagents employed were of analytical grade and used without further purification. Human plasma was obtained from Wuhan Third Hospital. Ultrapure water, obtained from a Millipore water purification system (18.2 MΩ·cm resistivity, Milli-Q Direct 8), was used in all runs.

### 3.2. Apparatus and Measurements

Firstly, the H1, H2, and H3 strands were heated at 95 °C for 5 min, followed by slowly cooling them down to room temperature. Secondly, different concentrations of PSA were mixed with the H1 solution in working buffer (10 mM Tris-HCl, 100 mM NaCl, pH 7.4) at 37 °C. After 30 min, the H2 and H3 were added and incubated at 37 °C. Then, GelRed (1 × 500) was added to the mixture. Finally, GO was added, and the mixture solution was vortexed. After 3 min, the solution was diluted to 1 mL, and the fluorescence intensity of the solutions were measured.

The fluorescence spectra were obtained with a Hitachi F-4600 spectrophotometer (Hitachi Co. Ltd., Tokyo, Japan, www.hitachi.co.jp) equipped with a xenon lamp excitation source at room temperature. The excitation was set at 518 nm, and the emission spectra ranged from 580–750 nm. The slits of the excitation and emission were set at 5 nm. The control experiments had the same measurement process, except without the addition of PSA. At the same time, each experiment was repeated 3 times, and the relative standard deviations were plotted as the error bar. In addition, three different potential interference compounds, CD63, BSA, and D-dimer, with a concentration of 50 ng/mL, were added with the absence, as well as presence, of 5 ng/mL PSA, to investigate the selectivity and specificity of the established assay. The value of the fluorescence intensity was used for quantitative assay of PSA. F_1_ and F_0_ is the fluorescence values at 606 nm in the presence and absence of PSA, respectively.

## 4. Conclusions

In summary, an enzyme-free and label-free fluorescence bioassay, based on GO, HCR, and GelRed, has been developed for the detection of PSA in the present work. As an enzyme-free bioassay, it has a good adaptability to variable surroundings. Therefore, it can be used for the detection of PSA in different human bodily fluids. Moreover, GelRed as the signal probe, through a chelating action, can avoid the disadvantage of labeling. At the same time, the established assay platform has many other merits, such as a low detection limit, simplicity of operation, and an excellent specificity. More importantly, the applications in biological samples also indicate that this bioassay exhibits great potential in clinical and basic research.

## Figures and Tables

**Table 1:** Performance comparison of different methods.

Method	Linear Range	Detection Limit	Reference
Microfluidic Devices	10–100 ng/mL	10 ng/mL	[5]
Rresonance light scattering	0.13–110 ng/mL	0.13 ng/mL	[4]
Immunosensor	0.05–26 ng/mL	0.05 ng/mL	[30]
electrogenerated chemiluminescence immunosensor	8–10 pg/mL	8 pg/mL	[31]
quartz crystal microbalance sensor	0.29–150 ng/mL	0.29 ng/mL	[32]
Electrochemistry	0.05–100 ng/mL	1000 pg/mL	[33]
Field effect transistor	0.023–500 ng/mL	23 pg/mL	[34]
Fluoroimmunoassay	0.1–200 ng/mL	10 pg/mL	This Work

## Supplementary Materials

The Supplementary Materials are available online.
