# Supplementary material for: A Non-Enzymatic and Label-Free Fluorescence Bioassay for Ultrasensitive Detection of PSA
Source: Molecules. 2019 Feb 26;24(5):831. doi: 10.3390/molecules24050831 (PMC6429520; doi:10.3390/molecules24050831)
Supplement: Supplementary file 1 [file molecules-24-00831-s001.pdf]

A non-enzymatic and label-free fluoroimmunoassay for ultrasensitive detection of PSA

Yujie Sun[1#] ·Chenyun Wang[1#]· Linhai Wang[2]·Hong Zhang[3]·Yulin Zhang[*,1] ·Guojun Zhang[*, 1]

[1]School of Laboratory Medicine, Hubei University of Chinese Medicine, 1 Huangjia Lake West Road, Wuhan 430065, People's Republic of China

[2]Teaching and Research Office of Forensic Medicine, Hubei University of Chinese Medicine, 1 Huangjia Lake West Road, Wuhan 430065, China

1.Optimization of reaction conditions

The fluorescence signal in the presence of H2, H3 and gelred can be effectively quenched by GO is vital for the design of the HCR/GO/Gelred assay. There are a variety of factors can influence the sensitivity in this experiment. As shown in Fig. S1A, it can be seen that, the fluorescence intensity of F_1 and F_0 initially increased and then decreased with the increasing of pH, the maximum F_1/F_0 values was attained at pH 7.4. so the 7.4 was selected as the optimum pH value. Fig. S1B shows that the incubation temperature could obviously affect the sensitivity. To begin with, the fluorescence signal of F_1 and F_0 increased when the temperature rises and then decreased gradually over 37°C. The value of F_1, F_0, and F_1/F_0 reached the maximum when the incubation temperature was 37°C, so the 37°C was chosen as the HCR reaction temperature in the design experiments.

The concentration of H1, H2 and H3 were also investigated in order to obtain the high sensitivity. As shown in Fig. S2A, the F_1/F_0 value reached a maximum with 30 nM H2. 30 nM as the optimized concentration of H2 was therefore selected in the following experiments and the concentrations of H3 are identical with H2.

---

[*] Corresponding author: Tel: +86-27-68890259, Fax: +86-27-68890259
E-mail: zhanggj@hbtcm.edu.cn
[#]Yujie Sun and Chenyun Wang equally contributed to this work

As shown in Fig. S2B, the value of F_1 and F_0 are both always increasing with the the increase of H1 concentration. But the maximum F_1/F_0 value is observed with 20 nM H1. 20 nM was confirmed as the optimized reaction concentration of H1.

Fig. S1.(A)The effect of reaction PH on the fluorescence response of this method. (B) Fluorescence response at different reaction temperatures. Light yellow columns: control experiments; dark cyan columns: with 5 ng·mL-1 of target; both H2 and H3 are 30 nM. The black lines represent the F_1/F_0 at different conditions, while the F_1 and F_0 were the fluorescence intensities in the presence and absence of PSA, respectively.

Fig. S2.(A)The effect of H2 concentration on the fluorescence response of this assay. (B) Fluorescence response at different H1 concentration. The F_1 and F_0 were the fluorescence intensities in the presence and absence of 5 ng·mL^{-1} PSA, respectively.

Figure S3. The typical fluorescent emission spectra of different mixtures solution, the adding solution from curve a to d: (a)H1+GO; (b) H1+PSA+GO.

Figure S4. Fluorescence response of the assay for detection of PSA(0.5 ng/mL) in buffer and various blank biological samples (human serum, urine and saliva, respectively).

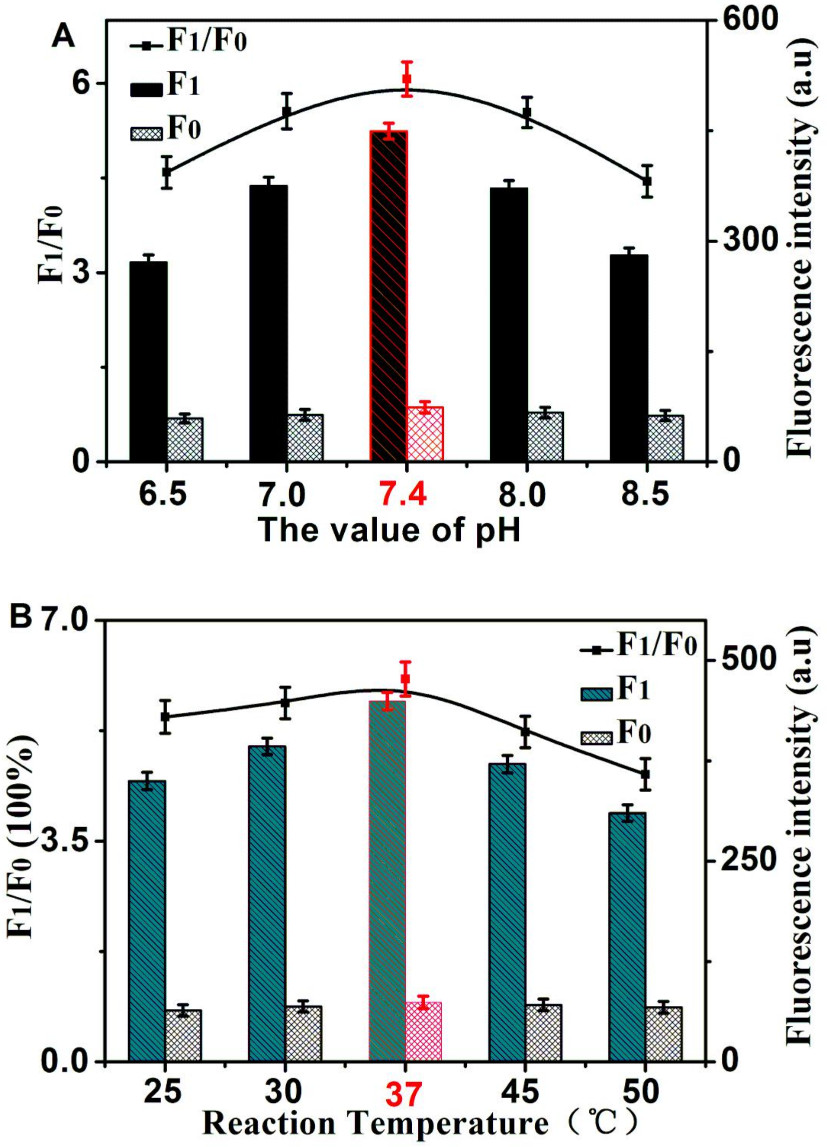

A
F1/F0
F1
F0
6
3
0
600
300
0
F1/F0
Fluorescence intensity (a.u)
6.5
7.0
7.4
8.0
8.5
The value of pH
B
7.0
3.5
0.0
500
250
0
F1/F0 (100%)
Fluorescence intensity (a.u)
25
30
37
45
50
Reaction Temperature（℃）

Figure S1

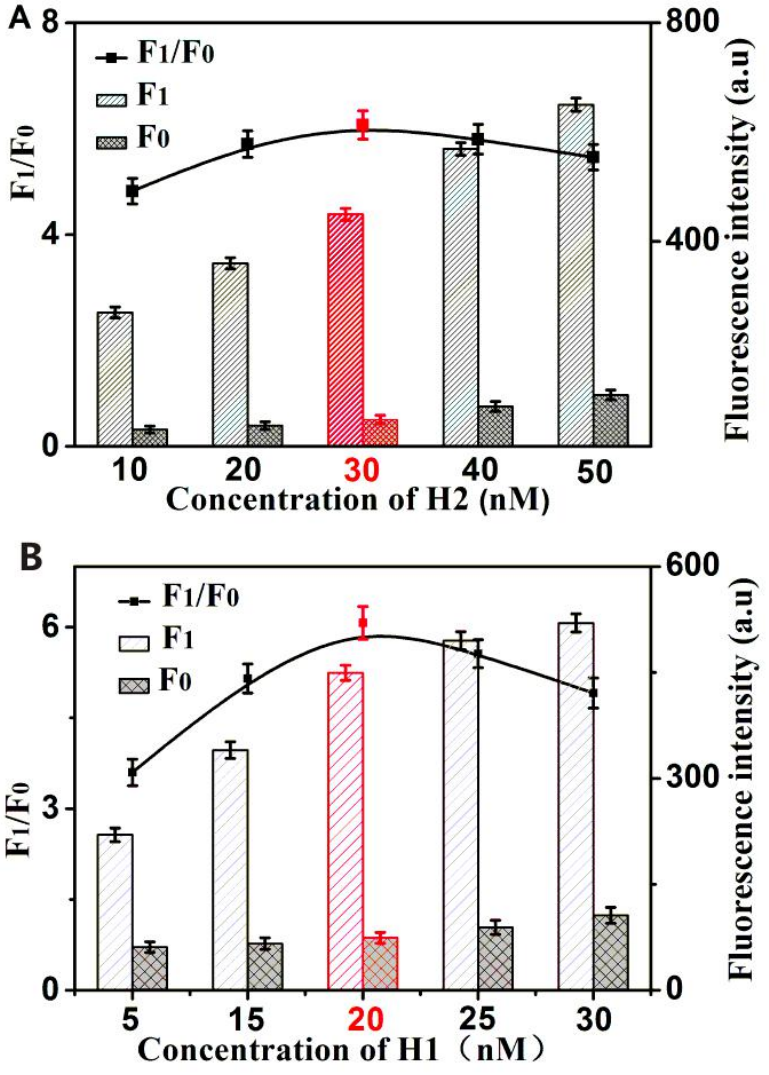
A
F1/F0
F1
F0
F1/F0
Fluorescence intensity (a.u)
0
4
8
0
400
800
10
20
30
40
50
Concentration of H2 (nM)
B
F1/F0
F1
F0
F1/F0
Fluorescence intensity (a.u)
0
3
6
0
300
600
5
15
20
25
30
Concentration of H1（nM）

Figure S2

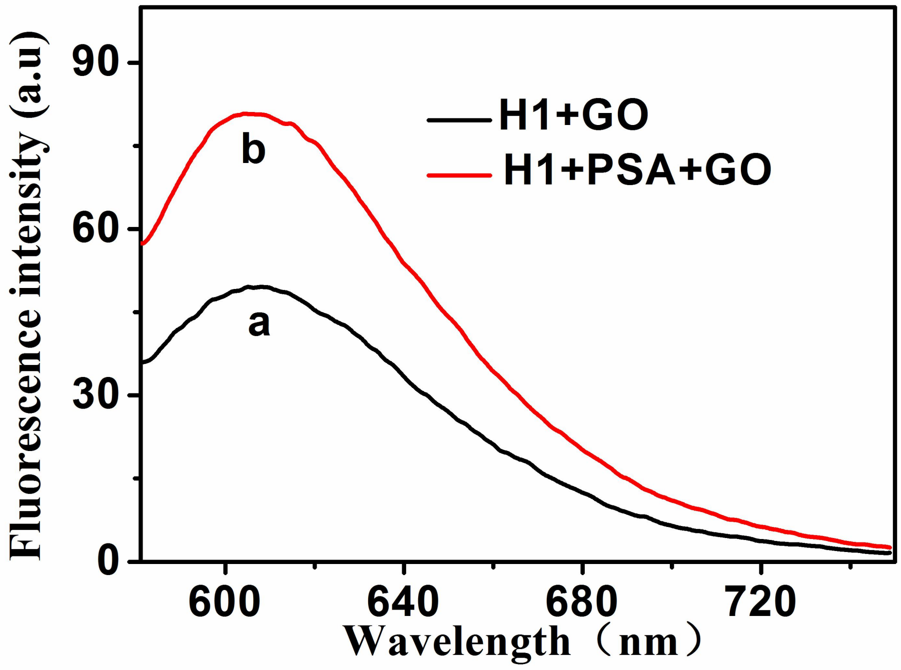

Figure S3

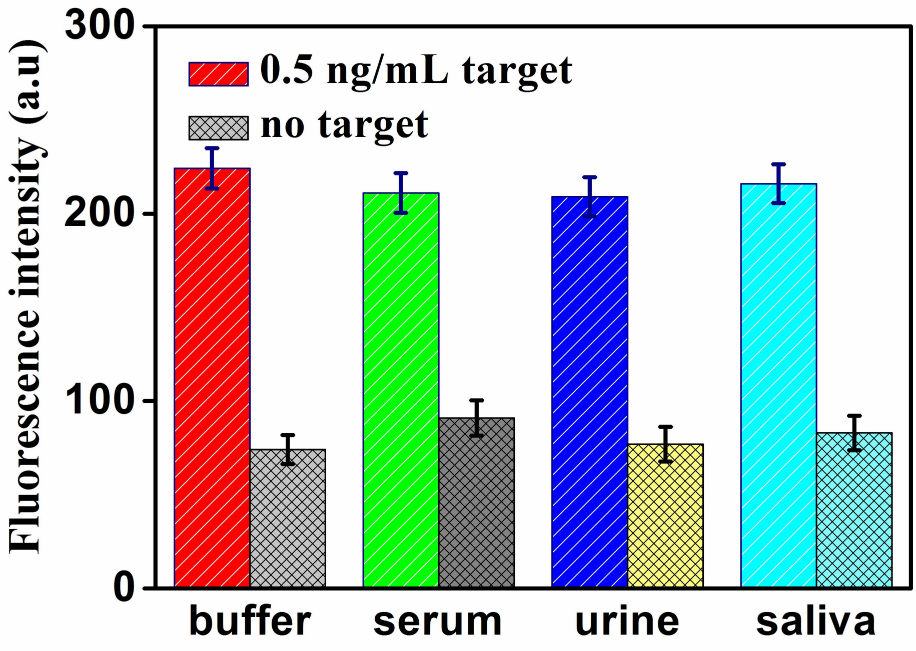

Figure S4
